# Associations between dental knowledge, source of dental knowledge and oral health behavior in Japanese university students: A cross-sectional study

**DOI:** 10.1371/journal.pone.0179298

**Published:** 2017-06-08

**Authors:** Ayano Taniguchi-Tabata, Daisuke Ekuni, Shinsuke Mizutani, Mayu Yamane-Takeuchi, Kota Kataoka, Tetsuji Azuma, Takaaki Tomofuji, Yoshiaki Iwasaki, Manabu Morita

**Affiliations:** 1 Department of Preventive Dentistry, Okayama University Graduate School of Medicine, Dentistry and Pharmaceutical Sciences, Okayama, Japan; 2 Advanced Research Center for Oral and Craniofacial Sciences, Okayama University Dental School, Okayama, Japan; 3 Section of Geriatric Dentistry, Department of General Dentistry, Fukuoka Dental College, Fukuoka, Japan; 4 Department of Community Oral Health, Asahi University School of Dentistry, Gifu, Japan; 5 Health Service Center, Okayama University, Okayama, Japan; Mexican Social Security Institute, MEXICO

## Abstract

The aim of this study was to investigate the associations between dental knowledge, the source of dental knowledge and oral health behavior in a group of students at a university in Japan. A total of 2,220 university students (1,276 males, 944 females) volunteered to undergo an oral examination and answer a questionnaire. The questionnaire assessed dental knowledge, the source of dental knowledge and oral health behavior (e.g., daily frequency of tooth brushing, use of dental floss and regular dental checkups). The odds ratio and 95% confidence interval for oral health behavior based on dental knowledge and source of dental knowledge were calculated using logistic regression models. Of the participants, 1,266 (57.0%) students obtained dental knowledge from dental clinics, followed by school (39.2%) and television (29.1%). Logistic regression analyses indicated that use of dental floss was significantly associated with source of dental knowledge from dental clinics (*P* = 0.006). Receiving regular dental checkups was significantly associated with source of dental knowledge; the positive source was dental clinic (*P* < 0.001) and the negative sources were school (*P* = 0.004) and television (*P* = 0.018). Dental clinic was the most common source of dental knowledge and associated with better oral health behavior among the Japanese university students in this study.

## Introduction

In research on major chronic diseases, more emphasis has been placed on the influence of health behavior rather than standard risk factors [1]. This concept has spread to the field of dentistry as well. In fact, oral diseases including periodontal disease can be prevented by adopting proper oral health behavior [2–4].

Proper oral health behavior such as tooth brushing, use of dental floss and receiving regular dental checkups prevents periodontal disease [5,6]. Furthermore, oral health behavior is also associated with various factors including dental knowledge [7–9], attitude [8–10], lifestyle [11–13], stress [14,15], education level [16], socioeconomic status [17,18], sense of coherence [19] and self-efficacy [5]. Among these factors, we focused on dental knowledge in a previous study and found that university students with dental knowledge practiced better oral health behavior such as use of dental floss [20].

University students are able to obtain dental knowledge through various means. For example, a television campaign as a source of dental knowledge demonstrated a significant impact on knowledge of periodontal health and disease in adults [21–23]. Another study also suggested that school is meaningful for oral health education of children as a source of dental knowledge [24]. Furthermore, dental knowledge from dental clinics may be effective at modifying oral health behavior [25–27]. Therefore, there may be effective sources of dental knowledge that contribute to oral health behavior or periodontal disease. However, few studies have reported the influence of various sources of dental knowledge on oral health behavior.

According to these results, we hypothesized that both the source of dental knowledge and dental knowledge itself correlate with oral health behavior in university students. The aim of this study was to investigate the associations between the source of dental knowledge, dental knowledge and oral health behavior in a group of students at a university in Japan.

## Materials and methods

### Study population

In April 2014, first-year students (n = 2,288) at Okayama University were invited in this study and 2,270 students received voluntary oral examinations and completed a questionnaire (response rate = 99.2%). The participants were recruited from 11 faculties (Faculties of Letters, Education, Law, Economics, Science, Pharmaceutical Sciences, Engineering, Environmental Science and Technology, Agriculture, Medicine and Dentistry). The inclusion criteria were young adults (18–24 years old), and students who received oral examinations and completed the questionnaire. The exclusion criteria were participants aged ≥ 25 years old and those who had provided incomplete responses in the questionnaire. Participants aged ≥ 25 years old (n = 25) and those who had provided incomplete responses in the questionnaire (n = 25) were excluded. Finally, data from 2,220 students (1,276 males, 944 females) were subjected to analysis.

Informed consent was obtained verbally from each participant. The protocol of this study was approved by the Ethics Committee of Okayama University Graduate School of Medicine, Dentistry and Pharmaceutical Sciences (No. 808).

### Questionnaire

A self-administered questionnaire was delivered by postal mail to each participant before dental examinations were conducted. In addition to sex and age, the questionnaire included the following items.

#### Dental knowledge

Questionnaire items asked participants whether they could explain dental terms, such as calculus, dental plaque, dental floss, sealant, periodontal disease, temporomandibular disorder, fluoride-containing mouthwash, topical application of fluoride and 8020 movement [20]. “8020 movement” is a Japanese social campaign aiming to retain 20 or more of one’s own teeth at the age of 80 [28]. Answers were given in a “yes/no” format.

#### Source of dental knowledge

Participants were also requested to state where they acquired dental knowledge. Participants were able to indicate up to three sources from the following: dental clinic, school, television, family, internet, newspaper, acquaintance and publication.

#### Oral health behavior

Participants were asked about the following oral health behavior: daily frequency of tooth brushing (≥ 2 times/≤ 1 time); use of dental floss (yes/no); and receiving regular dental checkups during the past year (yes/no) [5,6].

### Oral examination

Five dentists (D.E., A.T., S.M., M.Y-T. and K.K.) examined the participants’ periodontal status. The periodontal status was assessed using the Community Periodontal Index (CPI) [29] using a CPI probe (YDM, Tokyo, Japan) to evaluate six sites on each tooth (mesio-buccal, mid-buccal, disto-buccal, disto-lingual, mid-lingual and mesio-lingual). For periodontal examination, the following 10 teeth were selected: two molars in each posterior sextant and the upper right and lower left central incisors. As bleeding on probing (BOP) is an earlier and more sensitive indicator of gingival inflammation than the probing depth [30], the percentage of teeth exhibiting BOP (%BOP) among the ten examined teeth was also assessed [20]. The intra- and inter-examiner reliabilities, evaluated by κ statistics, of the CPI score were > 0.8.

#### Statistical analyses

The unpaired *t* test and chi-squared test were used to determine significant differences according to sex. Secondly, the chi-squared test was used to determine significant differences according to oral health behavior.

The odds ratio (OR) and 95% confidence interval (CI) were calculated using a logistic regression model. Each oral health behavior was considered a dependent variable. The multivariate analysis included sex, age, dental knowledge and the source of dental knowledge as independent variables. Independent variables were selected when the P value was <0.05 for the chi-square test or unpaired t-test in each variable.

Lastly, we evaluated the significant differences in oral health behavior between the two groups of periodontal statuses using the chi-squared test. The CPI and %BOP were divided into the following two groups: presence of probing pocket depth (PPD) ≥ 4 mm (CPI codes 0, 1 or 2 vs. CPI codes 3 or 4) or %BOP (< 20 vs. ≥ 20) [31].

All statistical analyses were performed using SPSS (version 22.0; IBM, Tokyo, Japan) with a level of significance set at *P* < 0.05.

## Results

Table 1 shows the differences in parameters between males and females. Males had worse oral health behavior than females (*P* < 0.01). Additionally, males had significantly less dental knowledge of the seven terms than females (*P* < 0.05). More than half of the students acquired dental knowledge from a dental clinic, followed by school and television. In contrast, newspaper, acquaintance and publication were rarely chosen as the source of dental knowledge.

**Table 1:** Differences in parameters between males and females.

		Males	Females	Total	*P* value ^a^
		n = 1,276	n = 944	n = 2,220	
**Age**		18.4 (0.7)	18.3 (0.7)	18.4 (0.7)	0.009
**Oral health behavior**					
Tooth brushing (daily frequency)	≥Two times	1,052 (82.4)	870 (92.2)	1,922 (86.6)	<0.001
	≤One time	224 (17.6)	74 (7.8)	298 (13.4)	
Use of dental floss	Yes	139 (10.9)	153 (16.2)	292 (13.2)	<0.001
Regular dental checkups	Yes	182 (14.3)	197 (20.9)	379 (17.1)	<0.001
**Dental knowledge**					
Dental plaque	Yes	433 (33.9)	347 (36.8)	780 (35.1)	0.168
Calculus	Yes	364 (28.5)	357 (37.8)	721 (32.5)	<0.001
Periodontal disease	Yes	278 (21.8)	269 (28.5)	547 (24.6)	<0.001
8020 movement	Yes	217 (17.0)	250 (26.5)	467 (21.0)	<0.001
Temporomandibular disorder	Yes	128 (10.0)	171 (18.1)	299 (13.5)	<0.001
Dental floss	Yes	83 (6.5)	115 (12.2)	198 (8.9)	<0.001
Topical application of fluoride	Yes	41 (3.2)	50 (5.3)	91 (4.1)	0.014
Fluoride-containing mouthwash	Yes	26 (2.0)	21 (2.2)	47 (2.1)	0.762
Sealant	Yes	15 (1.2)	25 (2.6)	40 (1.8)	0.010
**Source of dental knowledge**					
Dental clinic	Yes	687 (53.8)	579 (61.3)	1,266 (57.0)	<0.001
School	Yes	453 (35.5)	417 (44.2)	870 (39.2)	<0.001
Television	Yes	383 (30.0)	262 (27.8)	645 (29.1)	0.246
Family	Yes	287 (22.5)	204 (21.6)	491 (22.1)	0.621
Internet	Yes	289 (22.6)	136 (14.4)	425 (19.1)	<0.001
Newspaper	Yes	40 (3.1)	37 (3.9)	77 (3.5)	0.318
Acquaintance	Yes	52 (4.1)	23 (2.4)	75 (3.4)	0.035
Publication	Yes	36 (2.8)	19 (2.0)	55 (2.5)	0.226

Values are reported as mean (standard deviation) for age and number (percentage) for dichotomous variables.

^a^Compared males and females using *t* test or chi-square test.

There were no significant differences in dental knowledge and source of dental knowledge between the two groups of tooth brushing frequency (Table 2). There were significant differences in dental knowledge of calculus, dental floss and sealant according to use of dental floss and receiving regular dental checkups (*P* < 0.05). Participants who acquired dental knowledge from a dental clinic reported significantly greater use of dental floss and regular dental checkups than participants who did not (*P* < 0.05) However, participants who acquired dental knowledge from school or television reported significantly lower use of dental floss and regular dental checkups than participants who did not (*P* < 0.05).

**Table 2:** Association between oral health behavior and dental knowledge or source of dental knowledge.

		Tooth brushing (daily frequency)		Use of dental floss		Regular dental checkups	
		≥Two times	≤One time	Yes	No	Yes	No
		n = 1,922	n = 298	n = 292	n = 1,928	n = 379	n = 1,841
**Dental knowledge**							
Dental plaque	Yes	682 (35.5)	98 (32.9)	126 (43.2)**	654 (33.9)	138 (36.4)	642 (34.9)
Calculus	Yes	635 (33.0)	86 (28.9)	123 (42.1)**	598 (31.0)	173 (45.6)**	548 (29.8)
Periodontal disease	Yes	481 (25.0)	66 (22.1)	86 (29.5)*	461 (23.9)	103 (27.2)	444 (24.1)
8020 movement	Yes	406 (21.1)	61 (20.5)	60 (20.5)	407 (21.1)	84 (22.2)	383 (20.8)
Temporomandibular disorder	Yes	266 (13.8)	33 (11.1)	37 (12.7)	262 (13.6)	59 (15.6)	240 (13.0)
Dental floss	Yes	171 (8.9)	27 (9.1)	57 (19.5)**	141 (7.3)	58 (15.3)**	140 (7.6)
Topical application of fluoride	Yes	80 (4.2)	11 (3.7)	18 (6.2)	73 (3.8)	25 (6.6)*	66 (3.6)
Fluoride-containing mouthwash	Yes	43 (2.2)	4 (1.3)	11 (3.8)*	36 (1.9)	12 (3.2)	35 (1.9)
Sealant	Yes	37 (1.9)	3 (1.0)	13 (4.5)**	27 (1.4)	17 (4.5)**	23 (1.2)
**Source of dental knowledge**							
Dental clinic	Yes	1,104 (57.4)	162 (54.4)	201 (68.8)**	1,065 (55.2)	301 (79.4)**	965 (52.4)
School	Yes	752 (39.1)	118 (39.6)	98 (33.6)*	772 (40.0)	115 (30.3)**	755 (41.0)
Television	Yes	549 (28.6)	96 (32.2)	69 (23.6)*	576 (29.9)	80 (21.1)**	565 (30.7)
Family	Yes	418 (21.7)	73 (24.5)	82 (28.1)**	409 (21.1)	91 (24.0)	400 (21.7)
Internet	Yes	363 (18.9)	62 (20.8)	64 (21.9)	361 (18.7)	62 (16.4)	363 (19.7)
Newspaper	Yes	70 (3.6)	7 (2.3)	9 (3.1)	68 (3.5)	11 (2.9)	66 (3.6)
Acquaintance	Yes	62 (3.2)	13 (4.4)	13 (4.5)	62 (3.2)	9 (2.4)	66 (3.6)
Publication	Yes	51 (2.7)	4 (1.3)	7 (2.4)	48 (2.5)	7 (1.8)	48 (2.6)

Values are reported as number (percentage).

*P < 0.05

**P < 0.01, chi-square test.

Regarding the association between oral health behavior and dental knowledge or source of dental knowledge, the parameters that showed the significant differences in oral health behavior were partially different between males and females (Tables 3 and 4).

**Table 3:** Association between oral health behavior and dental knowledge or source of dental knowledge in males.

		Tooth brushing (daily frequency)		Use of dental floss		Regular dental checkup	
		≥Two times	≤One time	Yes	No	Yes	No
		n = 1,052	n = 224	n = 139	n = 1,137	n = 182	n = 1,094
**Dental knowledge**							
Dental plaque	Yes	364 (34.6)	69 (30.8)	63 (45.3)*	370 (32.5)	76 (41.8)*	357 (32.6)
Calculus	Yes	303 (28.8)	61 (27.2)	55 (39.6)*	309 (27.2)	84 (46.2)**	280 (25.6)
Periodontal disease	Yes	232 (22.1)	46 (20.5)	38 (27.3)	240 (21.1)	45 (24.7)	233 (21.3)
8020 movement	Yes	177 (16.8)	40 (17.9)	23 (16.5)	194 (17.1)	30 (16.5)	187 (17.1)
Temporomandibular disorder	Yes	109 (10.4)	19 (8.5)	17 (12.2)	111 (9.8)	22 (12.1)	106 (9.7)
Dental floss	Yes	65 (6.2)	18 (8.0)	18 (12.9)*	65 (5.7)	26 (14.3)**	57 (5.2)
Topical application of fluoride	Yes	35 (3.3)	6 (2.7)	6 (4.3)	35 (3.1)	11 (6.0)*	30 (2.7)
Fluoride-containing mouthwash	Yes	22 (2.1)	4 (1.8)	7 (5.0)*	19 (1.7)	8 (4.4) *	18 (1.6)
Sealant	Yes	14 (1.3)	1 (0.4)	3 (2.2)	12 (1.1)	5 (2.7)*	10 (0.9)
**Source of dental knowledge**							
Dental clinic	Yes	569 (54.1)	118 (52.7)	92 (66.2)*	595 (52.3)	135 (74.2)**	552 (50.5)
School	Yes	369 (35.1)	84 (37.5)	44 (31.7)	409 (36.0)	57 (31.3)	396 (36.2)
Television	Yes	313 (29.8)	70 (31.3)	36 (25.9)	347 (30.5)	38 (20.9)*	345 (31.5)
Family	Yes	230 (21.9)	57 (25.4)	36 (25.9)	251 (22.1)	41 (22.5)	246 (22.5)
Internet	Yes	238 (22.6)	51 (22.8)	34 (24.5)	255 (22.4)	40 (22.0)	249 (22.8)
Newspaper	Yes	35 (3.3)	5 (2.2)	4 (2.9)	36 (3.2)	7 (3.8)	33 (3.0)
Acquaintance	Yes	40 (3.8)	12 (5.4)	9 (6.5)	43 (3.8)	4 (2.2)	48 (4.4)
Publication	Yes	33 (3.1)	3 (1.3)	5 (3.6)	31 (2.7)	5 (2.7)	31 (2.8)

Values are reported as number (percentage).

*P < 0.05

**P < 0.01, chi-square test.

**Table 4:** Association between oral health behavior and dental knowledge or source of dental knowledge in females.

		Tooth brushing (daily frequency)		Use of dental floss		Regular dental checkup	
		≥Two times	≤One time	Yes	No	Yes	No
		n = 870	n = 74	n = 153	n = 791	n = 197	n = 747
**Dental knowledge**							
Dental plaque	Yes	318 (36.6)	29 (39.2)	63 (41.2)	284 (35.9)	62 (31.5)	285 (38.2)
Calculus	Yes	332 (38.2)	25 (33.8)	68 (44.4)	289 (36.5)	89 (45.2)*	268 (35.9)
Periodontal disease	Yes	249 (28.6)	20 (27.0)	48 (31.4)	221 (27.9)	58 (29.4)	211 (28.2)
8020 movement	Yes	229 (26.3)	21 (28.4)	37 (24.2)	213 (26.9)	54 (27.4)	196 (26.2)
Temporomandibular disorder	Yes	157 (18.0)	14 (18.9)	20 (13.1)	151 (19.1)	37 (18.8)	134 (17.9)
Dental floss	Yes	106 (12.2)	9 (12.2)	39 (25.5)**	76 (9.6)	32 (16.2)	83 (11.1)
Topical application of fluoride	Yes	45 (5.2)	5 (6.8)	12 (7.8)	38 (4.8)	14 (7.1)	36 (4.8)
Fluoride-containing mouthwash	Yes	21 (2.4)	0 (0)	4 (2.6)	17 (2.1)	4 (2.0)	17 (2.3)
Sealant	Yes	23 (2.6)	2 (2.7)	10 (6.5)*	15 (1.9)	12 (6.1)*	13 (1.7)
**Source of dental knowledge**							
Dental clinic	Yes	535 (61.5)	44 (59.5)	109 (71.2)*	470 (59.4)	166 (84.3)**	413 (55.3)
School	Yes	383 (44.0)	34 (45.9)	54 (35.3)*	363 (45.9)	58 (29.4)**	359 (48.1)
Television	Yes	236 (27.1)	26 (35.1)	33 (21.6)	229 (29.0)	42 (21.3)*	220 (29.5)
Family	Yes	188 (21.6)	16 (21.6)	46 (30.1)*	158 (20.0)	50 (25.4)	154 (20.6)
Internet	Yes	125 (14.4)	11 (14.9)	30 (19.6)*	106 (13.4)	22 (11.2)	114 (15.3)
Newspaper	Yes	35 (4.0)	2 (2.7)	5 (3.3)	32 (4.0)	4 (2.0)	33 (4.4)
Acquaintance	Yes	22 (2.5)	1 (1.4)	4 (2.6)	19 (2.4)	5 (2.5)	18 (2.4)
Publication	Yes	18 (2.1)	1 (1.4)	2 (1.3)	17 (2.1)	2 (1.0)	17 (2.3)

Values are reported as number (percentage).

*P < 0.05

**P < 0.01, chi-square test.

In logistic regression analyses, daily frequency of tooth brushing was significantly associated with sex (*P* < 0.001) (Table 5). Use of dental floss was significantly associated with sex (*P* = 0.004); having knowledge of dental floss (*P* < 0.001) and sealant (*P* = 0.048); and dental clinic as the source of dental knowledge (*P* = 0.006). Regular dental checkups were significantly associated with sex (*P* = 0.006), having knowledge about dental plaque (*P* = 0.008), calculus (*P* < 0.001), dental floss (*P* = 0.016) and sealant (*P* = 0.008), and the source of dental knowledge (*P* < 0.05). The positive source was dental clinic (*P* < 0.001) and the negative sources were school (*P* = 0.004) and television (*P* = 0.018).

**Table 5:** Adjusted OR and 95% CIs for oral health behavior.

		Tooth brushing (daily frequency)			Use of dental floss			Regular dental checkups		
		≥Two times per day			Yes			Yes		
		OR	95% CI	*P* value	OR	95% CI	*P* value	OR	95% CI	*P* value
**Age**		1.02	0.85–1.21	0.856	1.08	0.92–1.26	0.362	0.98	0.84–1.15	0.789
**Sex**	Male	1.00			1.00			1.00		
	Female	2.52	1.90–3.34	<0.001	1.45	1.12–1.87	0.004	1.39	1.10–1.75	0.007
**Dental knowledge**										
Dental plaque	No	1.00			1.00			1.00		
	Yes	1.09	0.80–1.48	0.597	1.13	0.84–1.52	0.418	0.68	0.51–0.90	0.008
Calculus	No	1.00			1.00			1.00		
	Yes	1.08	0.79–1.49	0.614	1.19	0.88–1.61	0.259	1.93	1.47–2.53	<0.001
Periodontal disease	No	1.00			1.00			1.00		
	Yes	1.08	0.77–1.50	0.656	0.96	0.70–1.32	0.815	0.88	0.65–1.17	0.379
Dental floss	No	1.00			1.00			1.00		
	Yes	0.75	0.47–1.19	0.219	2.30	1.58–3.35	<0.001	1.58	1.09–2.30	0.016
Topical application of fluoride	No	1.00			1.00			1.00		
	Yes	0.90	0.45–1.82	0.773	0.83	0.46–1.51	0.543	1.16	0.67–1.99	0.600
Fluoride-containing mouthwash	No	1.00			1.00			1.00		
	Yes	1.68	0.57–4.94	0.345	1.55	0.73–3.29	0.250	1.22	0.58–2.57	0.594
Sealant	No	1.00			1.00			1.00		
	Yes	1.67	0.50–5.55	0.406	2.06	1.01–4.20	0.048	2.53	1.27–5.03	0.008
**Source of dental knowledge**										
Dental clinic	No	1.00			1.00			1.00		
	Yes	1.02	0.79–1.32	0.868	1.47	1.12–1.94	0.006	2.92	2.22–3.85	<0.001
School	No	1.00			1.00			1.00		
	Yes	0.88	0.68–1.14	0.319	0.79	0.60–1.03	0.083	0.69	0.53–0.89	0.004
Television	No	1.00			1.00			1.00		
	Yes	0.84	0.64–1.11	0.219	0.78	0.58–1.06	0.110	0.71	0.54–0.94	0.018
Family	No	1.00			1.00			1.00		
	Yes	0.83	0.62–1.11	0.219	1.30	0.97–1.73	0.077	0.98	0.74–1.29	0.872

Adjusted for age, sex, dental knowledge and source of dental knowledge.

The association between oral health behavior and periodontal status is shown in S1 Table. Participants with low %BOP scores (< 20%) had significantly higher frequencies of using dental floss and regular dental checkups than participants with higher %BOP scores (≥ 20%) (*P* < 0.001) (S1 Table).

## Discussion

In this study, we focused on the associations between the source of dental knowledge, dental knowledge and oral health behavior. We found that differences in the source of dental knowledge were associated with oral health behavior in university students; that is, both positive and negative associations between the sources and oral health behavior were observed.

The source of dental knowledge from dental clinics contributed to good oral health behavior, i.e., use of dental floss and regular dental checkups. A previous study found that oral health education at dental clinics was effective at modifying oral health behavior [25–27], which was confirmed by our results. Since dentists are important sources of oral disease prevention for the general public [32], dental clinics could be the most effective location for university students to improve oral health behavior.

It is interesting to note that school and television were negatively associated with regular dental checkups (Table 5). Previous studies found that oral health education in primary or secondary school could improve oral health behavior [33–39]. On the other hand, other studies in school-based education programs found no improvement [40,41]. A mass media health education campaign on television could not demonstrate a significant impact on behavior [42,43]. Furthermore, a recent study suggests that oral health program without repetition could transiently improve oral health behavior, but could not sustain improved oral health behavior in the long-term [44]. Thus, oral health education should be repeated with either method to keep its positive results [41,44,45]. Taken together, the other sources except for dental clinics might not encourage regular dental checkups repeatedly.

A previous study found that oral health education from school teachers or dentists was equally effective in improving oral health knowledge and the oral hygiene status of adolescents [46]. However, the outcome of oral health education programs is dependent on the teachers’ instructions or motivations [47–49]. This finding may support our result that obtaining dental knowledge from school was negatively associated with receiving regular dental checkups.

On the other hand, multifaceted interventions such as education programs, with a combination of methods (lectures and small-group discussions), improved knowledge, skills and attitude compared to single interventions or no interventions [50]. In terms of an education program, “television”, “family”, “internet”, “newspaper”, “acquaintance” or “publication” involved no intervention, but “school” can have multifaceted interventions. This study demonstrated that school was one of the major sources of dental knowledge. However, school was negatively associated with oral health behavior. Therefore, more effective intervention to promote better oral health behavior among students should be introduced in the Japanese education curriculum.

In this study, the most common source of dental knowledge was dental clinic, followed by school. However, approximately 20 years ago, schools were the most common source of dental knowledge in Japan [24]. In other countries, more than 60% of adults obtained dental knowledge from dentists or dental clinics [32, 51]. Furthermore, in the United States and other western countries, adults visited the dental clinic more often [52–54]. In Japan, the rate of regular dental checkups has been increasing [55]. Thus, the source of dental knowledge may change from school to dental clinics.

The percentage of participants who could explain dental floss was different from that of participants who used dental floss. The previous study shows that the percentage of participants who comprehend dental floss was different to that of participants who used dental floss [56]. Thus, explainable level of dental knowledge might not completely imply oral health behavior. On the other hand, dental knowledge of dental floss was positively associated with use of dental floss, which was supported by the previous study [56]. As the relationship between knowledge and behavior can be complex, further studies are needed.

In our study, use of dental floss and receiving regular dental checkups were associated with %BOP (S1 Table); that is, participants with good oral health behavior had good periodontal status. This result supports the findings of a previous study [6]. Thus, acquiring dental knowledge from dental clinics may effectively induce good oral health behavior, which contributes to achieving and maintaining good periodontal status.

There are some limitations associated with our study. First, a causal association could not be shown because the study was cross-sectional. Second, other possible confounders, such as attitude [9–10], lifestyle [11–13], stress [14,15], education level [16], socioeconomic status [17,18], sense of coherence [19] and self-efficacy [5], were not included in this study. Third, we did not investigate the effects of frequency of obtaining information from the source, interaction between the assessed knowledge and the sources, frequency of dental floss use, the recall interval for the dental checkups, the experience and pattern of dental visitation, or the relation between oral health status and dental visitation pattern, which might affect oral health behavior. Finally, all participants were recruited from Okayama University students. It may limit the ability to extrapolate these findings to the general young population.

## Conclusion

The source of dental knowledge from dental clinics as well as having dental knowledge were associated with better oral health behavior in university students in Japan.

## Supporting information

S1 Table Association between oral health behavior and periodontal status. (DOCX)
