# Supplementary material for: Associations between dental knowledge, source of dental knowledge and oral health behavior in Japanese university students: A cross-sectional study
Source: PLoS One. 2017 Jun 8;12(6):e0179298. doi: 10.1371/journal.pone.0179298 (PMC5464648; doi:10.1371/journal.pone.0179298)
Supplement: S1 Table — (DOCX) [file pone.0179298.s001.docx]

**S1 Table. Association between oral health behavior and periodontal status.**

|  | **Presence of PPD ≥ 4 mm** | | | **%BOP** | | | |
| --- | --- | --- | --- | --- | --- | --- | --- |
|  | **(-)** | **(+)** |  | **< 20** | **≥ 20** |  | |
|  | **n=1,880** | **n=340** | ***P* value ^a^** | **n=741** | **n=1,479** | ***P* value ^b^** | |
| Tooth　brushing　(daily frequency) | | | | | | | |
| ≥Two times | 1,636 (86.3) | 299 (87.9) | 0.423 | 647 (87.3) | 1,275 (86.2) | 0.470 | |
| ≤One time | 257 (13.7) | 41 (12.1) |  | 94 (12.7) | 204 (13.8) |  | |
| Use of dental floss | | | | | | | |
| Yes | 256 (13.6) | 36 (10.6) | 0.128 | 129 (17.4) | 163 (11.0) | <0.001 | |
| No | 1,642 (86.4) | 304 (89.4) |  | 612 (82.6) | 1,316 (89.0) |  | |
| Regular dental checkups | | | | | | | |
| Yes | 312 (16.6) | 67 (19.7) | 0.161 | 166 (22.4) | 213 (14.4) | <0.001 | |
| No | 1,568 (83.4) | 273 (80.3) |  | 575 (77.6) | 1266 (85.6) |  | |
| PPD, probing pocket depth; BOP, bleeding on probing. | | | | | | |  |
| Values are reported as number (percentage). | | | | | | |  |
| ^a^Compared presence of PPD ≥ 4 mm (-) and (+) groups using chi-square test. | | | | | | |  |
| ^b^Compared %BOP < 20 and ≥ 20 groups using chi-square test. | | | | | | |  |
